# KINLESS OLDER ADULTS WITH DEMENTIA: QUALITATIVE ANALYSIS OF DATA FROM THE ADULT CHANGES IN THOUGHT STUDY

**DOI:** 10.1093/geroni/igae098.1190

**Published:** 2024-12-31

**Authors:** Janelle Taylor, Marlaine Figueroa Gray, Callie Freitag, Hitomi Kariya, Paul Crane, Elizabeth Vig, Clara Berridge, Eric Larson

**Affiliations:** University of Toronto, Toronto, Ontario, Canada; Kaiser Permanente Washington Health Research Institute (KPWHRI), Seattle, Washington, United States; University of Wisconsin-Madison, Wisconsin, United States; NA, Seattle, Washington, United States; University of Washington, Seattle, Washington, United States; University of Washington/VA Puget Sound Healthcare System, Seattle, Washington, United States; University of Washington, Seattle, Washington, United States; University of Washington, Seattle, Washington, United States

## Abstract

**Objectives:**

To examine the circumstances and needs of older adults who were “kinless,” defined as having no living spouse or children, when they developed dementia.

**Methods:**

We conducted a secondary analysis of information from the Adult Changes in Thought study. Among 848 participants diagnosed with dementia between 1994 and 2016, we identified 64 who had no living spouse or child at dementia onset. We then conducted a qualitative analysis of administrative documents pertaining to these participants: handwritten comments recorded after each study visit, and medical history documents containing clinical chart notes from participants’ medical records.

**Results:**

In this community-dwelling cohort of older adults diagnosed with dementia, 8.4% were kinless at dementia onset. Participants in this sample had an average age of 87 years old, half lived alone, and one third lived with unrelated persons. Through inductive content analysis, we identified 4 themes that describe their circumstances and needs: (1) life trajectories, (2) caregiving resources, (3) care needs and gaps, and (4) turning points in caregiving arrangements.

**Discussion:**

Our qualitative analysis reveals that the life trajectories that led members of the analytic cohort to be kinless at dementia onset were quite varied. This research highlights the importance of nonfamily caregivers and participants’ own roles as caregivers. Our findings suggest that clinicians and health systems may need to work with other parties to directly provide dementia caregiving support rather than rely on family, and address factors such as neighborhood affordability that particularly affect older adults who have limited family support.

